# The growth scale and kinetics of WS_2_ monolayers under varying H_2_ concentration

**DOI:** 10.1038/srep13205

**Published:** 2015-08-17

**Authors:** Kyung Nam Kang, Kyle Godin, Eui-Hyeok Yang

**Affiliations:** 1 Department of Mechanical Engineering, Stevens Institute of Technology, Hoboken, New Jersey 07030.

## Abstract

The optical and electronic properties of tungsten disulfide monolayers (WS_2_) have been extensively studied in the last few years, yet growth techniques for WS_2_ remain behind other transition metal dichalcogenides (TMDCs) such as MoS_2_. Here we demonstrate chemical vapor deposition (CVD) growth of continuous monolayer WS_2_ films on mm^2^ scales and elucidate effects related to hydrogen (H_2_) gas concentration during growth. WS_2_ crystals were grown by reduction and sulfurization of WO_3_ using H_2_ gas and sulfur evaporated from solid sulfur powder. Several different growth formations (in-plane shapes) were observed depending on the concentration of H_2_. Characterization using atomic force microscopy (AFM) and scanning electron microscopy (SEM) revealed etching of the SiO_2_ substrate at low concentrations of H_2_ and in the presence of an Ar carrier gas. We attribute this to insufficient reduction of WO_3_ during growth. High H_2_ concentrations resulted in etching of the grown WS_2_ crystals after growth. The two dimensional X-ray diffraction (2D XRD) pattern demonstrates that the monolayer WS_2_ was grown with the (004) plane normal to the substrate, showing that the WS_2_ conforms to the growth substrate.

In recent years, various monolayer transition metal dichalcogenides (TMDCs) have been studied as alternatives to graphene^1^,^2^,^3^,^4^,^5^. An isolated “monolayer” consists of a metal layer between two chalcogen layers and that S-W-S trilayer is bonded to neighboring layers through weak van der Waals bonds. While graphene has no band gap, TMDCs can be direct band gap semiconductors, which suggests many applications. Inheriting experimental techniques from graphene, circuit elements were quickly demonstrated in molybdenum disulfide (MoS_2_)^4^,^6^,^7^. Subsequently new properties of TMDCs have been discovered which drive their own applications, separate from its graphene ancestry, such as spintronics in MoS_2_^8^ and WSe_2_^9^ and tuning of the band gap through uniaxial strain^10^. Stacks of different TMDCs have been shown to create almost a novel material through the creation of new shared exciton states^11^ and through femtosecond scale charge separation^12^. TMDCs have also been explored for photodetectors, optical modulators, bio-imaging devices, mode-locked lasers, ultrafast saturation, and solar cells by using TMDCs alone or in graphene/TMDC heterostructures^13^,^14^,^15^,^16^,^17^,^18^.

WS_2_ has been less investigated than MoS_2_ simply because of the greater difficulty in producing samples through exfoliation. WS_2_, like MoS_2_, is of interest for optoelectronics because of its direct band gap in the visible range and high absorption relative to its thickness^19^,^20^. WS_2_ monolayers have strong PL emission, stronger than other TMDCs such as MoS_2_^21^. WS_2_ also exhibits strong spin-orbit coupling and band splitting due to spin enabling spintronics/valleytronics, which was first demonstrated in MoS_2_^22^,^23^,^24^. WS_2_ also has high nonlinear susceptibility, suggesting its use for nonlinear optical devices^25^,^26^. However, most device research to date has been largely based on mechanically exfoliated layers which does not allow high throughput manufacturing. Large scale deposition (polycrystalline chip scale or wafer scale growth, similar to what has already been demonstrated in graphene^27^ and MoS_2_^28^) and large grain size WS_2_ monolayers are essential for further application research and eventual commercialization. To date, CVD crystal growth of WS_2_ has been shown to produce single crystal flakes hundreds of micrometers in size^29^.

Here we demonstrate polycrystalline WS_2_ monolayer growth up to mm^2^ coverage and show the effect of H_2_ concentration on the crystal size, nucleation density, total areal density, and growth formation. Growth is performed by low pressure (LP) CVD from solid WO_3_ and S sources. We utilize Raman spectroscopy, optical microscopy, scanning electron microscopy (SEM), atomic force microscopy (AFM) and two dimensional X-ray diffraction (2D XRD) to characterize growth formation, crystallinity, substrate orientation, existence of monolayer growth, and height of the deposited WS_2_ layers. We furthermore demonstrate etching of WS_2_ or etching of the SiO_2_ substrate and explain these two effects in terms of the reaction chemistry. Finally, we report on a newly observed growth mode for single-crystal WS_2_ monolayers and propose a sequential growth model to explain our observations.

## Results and Discussion

**Figure 1:**
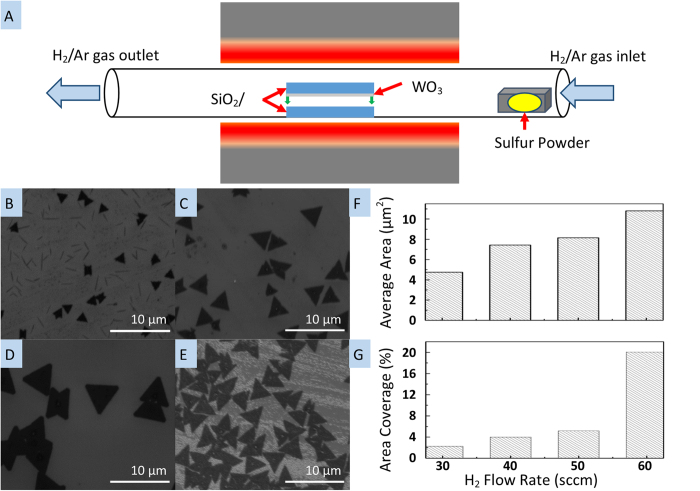
Schematic of the LPCVD setup for large-area WS_2_ deposition and SEM images of grown WS_2_ under H_2_/Ar mixture gas. The black triangle shapes are monolayer WS_2_. (**a**) Experimental setup. (**b**) as grown WS_2_ under 30 sccm H_2_ and 100 sccm Ar. (**c**) as grown WS_2_ under 40 sccm H_2_ and 100 sccm Ar. (**d**) as grown WS_2_ under 50 sccm H_2_ and 100 sccm Ar. (**e**) as grown WS_2_ under 60 sccm H_2_ and 100 sccm Ar. (**f**) graph of the average size of WS_2_ based on different H_2_ flow rates. (**g**) graph of the coverage of WS_2_ based on different H_2_ flow rates. All scale bars are 10 μm.

Figure 1a is an illustration of our experimental setup. The experiment was contained within a 3′′ diameter quartz tube with a mechanical pump at one end and gas (H_2_ and Ar) introduced at the other with a controlled flow rate. The operating pressure during growth was approximately 9 Torr with the H_2_/Ar mixture gas and 5 Torr with only H_2_ gas delivered. The center of the tube rests in a furnace which was raised to 900 °C and placed in the tube were two stacked substrates facing each other, one for the tungsten (W) source (WO_3_ evaporated by electron beam onto silicon) and for growth (an oxidized silicon substrate). Sulfur was provided by evaporation from solid powder which was placed in a ceramic crucible outside of the central furnace region, at a lower temperature. For large area growth, the sulfur position was such that it starts to evaporate when the substrate is between 800 and 850 °C. Hydrogen was introduced at 650 °C. Growth proceeded for thirty minutes, after which the furnace was cooled and hydrogen delivery ceased.

Figure 1b–e are SEM images of several WS_2_ monolayers grown with 30, 40, 50 and 60 sccm of H_2_ gas with 100 sccm Ar each. We note that there was no WS_2_ deposition when H_2_ was not introduced, but small, single crystal WS_2_ triangles were observed when the H_2_/Ar gas mixture was provided. The images show that when the flow rate of H_2_ was increased, both the size and density of crystals increased. Fig. 1f,g show the increasing size and total surface coverage measured by image analysis using ImageJ. The average size of a WS_2_ crystal increased from 4.7 μm^2^ to 10.8 μm^2^ over the experimental range, and total area coverage increased from 2.2% to 20%. The increase in total area coverage is not only due to increase in crystal size, but also due to increase in nucleation density, which increased approximately four times, from one nucleation site per 213 μm^2^ to one per 54 μm^2^ in the experimental range.

**Figure 2:**
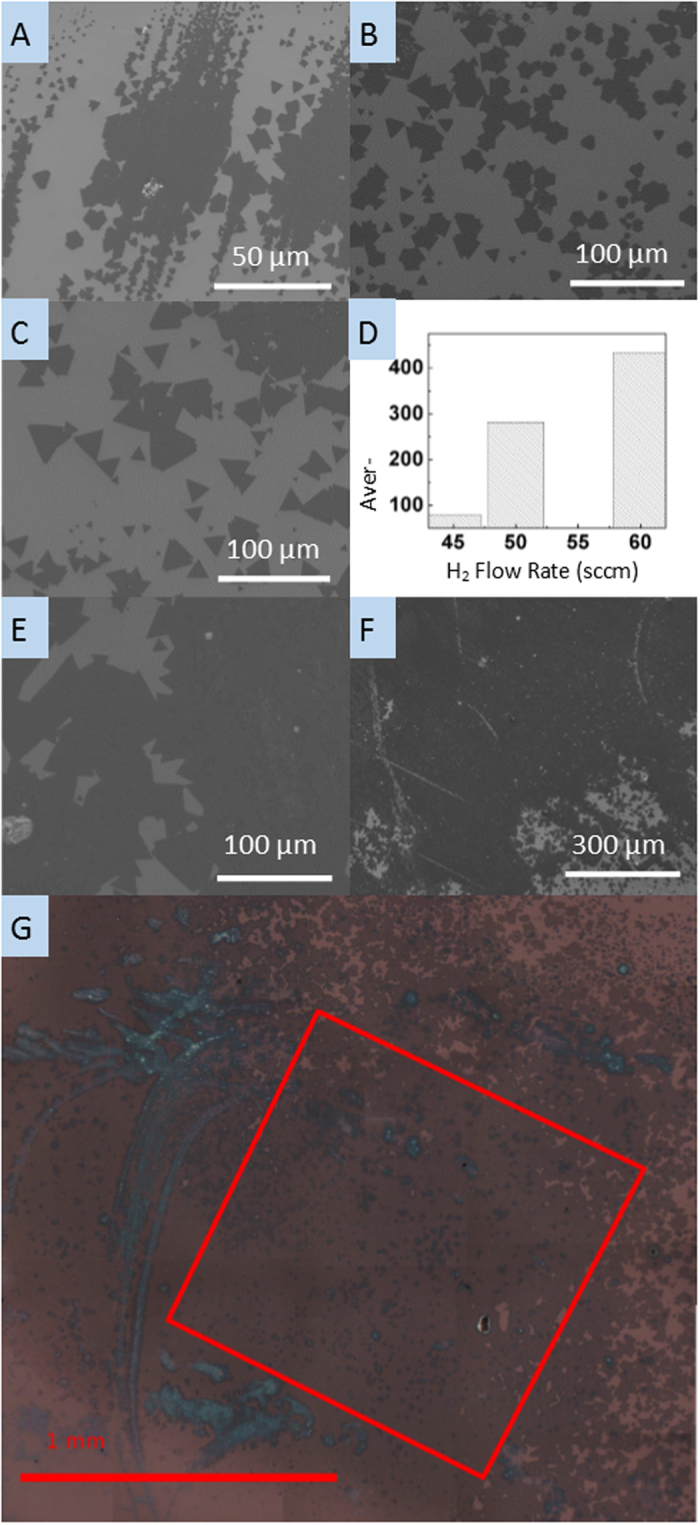
SEM images and optical image of grown WS_2_ under different H_2_ flow rates without Ar. The dark area is WS_2_ monolayer. (**a**) 45 sccm H_2_ flow rate. (**b**) 50 sccm. (**c**) 60 sccm. (**d**) graph of the average size of WS_2_ based on different H_2_ flow rates. (**e**) SEM image of polycrystalline WS_2_ grown under a 60 sccm H_2_ flow rate. (**f**) SEM image of a millimeter scale WS_2_ deposition. Scale bars are 100 μm except in (**f**) which is 300 μm. (**g**) is a stitched optical image of mm scale deposition; the red box is 1.1 mm square and monolayer covers 89.5% of the area within that region; scale bar 1mm.

Supplying H_2_ gas without Ar during the reduction and sulfurization process of WO_3_ resulted in increased single crystal size and nucleation density of WS_2_ monolayer crystals, compared to the same experimental conditions but with Ar. In addition to the increased size of single crystal WS_2_ monolayers, under certain conditions continuous mm^2^ coverage of polycrystalline WS_2_ monolayer growth was observed. Figures 2a–c show SEM images of WS_2_ monolayers grown with 45, 50 and 60 sccm of H_2_ gas and no Ar. Figure 2d shows the same trend as when Ar was present—when the H_2_ flow rate was increased the average size of the WS_2_ single-domain crystals increased. The crystals were larger than when Ar was present, from 79.8 μm^2^ under 45 sccm of H_2_ and up to 432.7 μm^2^ under 60 sccm. Figure 2e shows polycrystalline WS_2_ growth with more than 85% surface coverage, grown with 60 sccm H_2_. Figure 2f is a larger area SEM image of the polycrystalline growth, showing up to mm^2^ surface coverage. Each cm^2^ chip exhibited these large coverage regions along with regions of isolated single crystal growth such as the one used for the data in Fig. 2d. Figure 2g is a large-area optical image stitched to show the extent of large-area growth. The color contrast shows predominantly monolayer growth along with bilayer and thick growth in some regions. Within the red box, a 1.1 mm square, the monolayer coverage is 89.5%, calculated through thresholding and pixel counting performed with Gimp and ImageJ.

**Figure 3:**
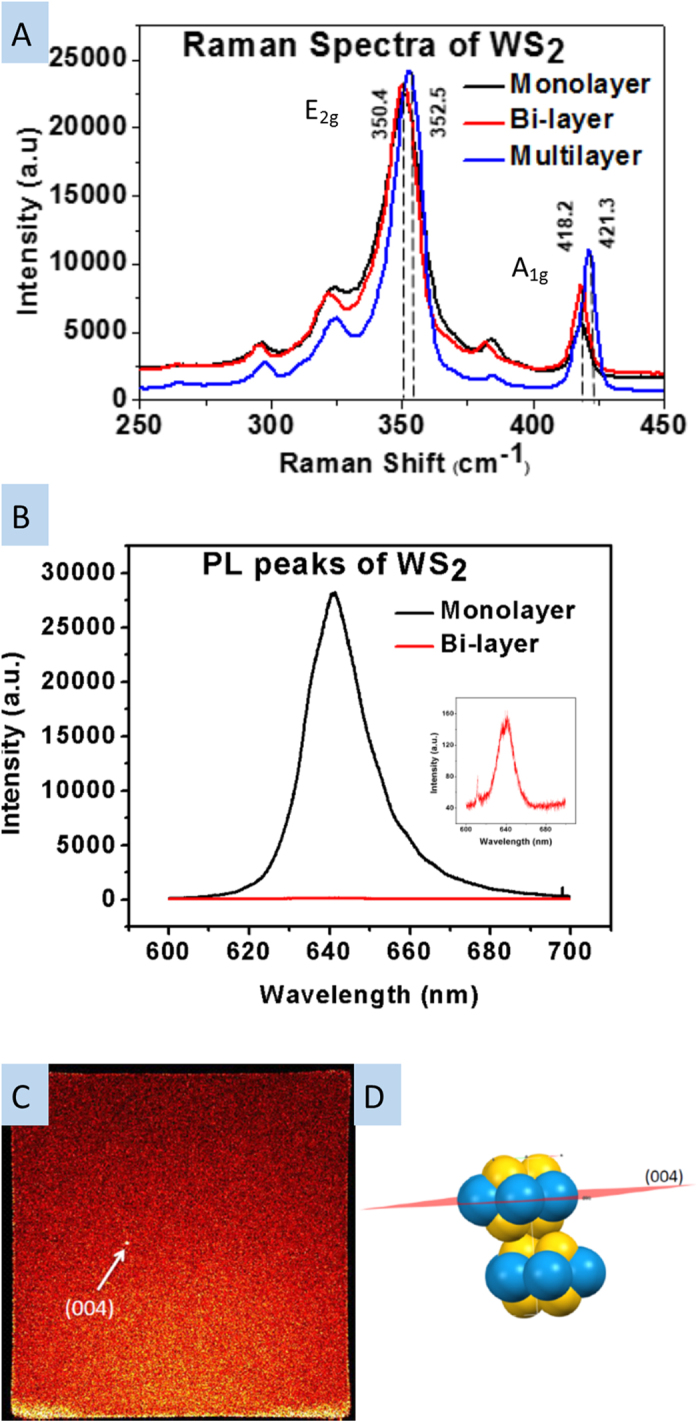
Raman spectroscopy of mono, bi and multi-layer as grown WS_2_. (**a**) Raman spectra of mono, bi and multi-layer WS_2_. (**b**) PL spectra of mono and bi-layer WS_2_. Inset: the magnified PL peak of bilayer WS_2_. (**c**) A 2D XRD image of well crystallized WS_2_ showing a sharp peak from the (004) plane. (**d**) atomic structure of WS_2_, showing the (004) plane.

Figure 3a,b show the Raman spectroscopy and photoluminescence (PL) peaks of mono, bi, and few layer WS_2_ samples grown in our furnace. All spectra were taken with 532 nm excitation. Figure 3a gives the E^1^_2g_ and A_1g_ phonon modes of mono, bi and multi-layer of WS_2_, located at 350.4 and 418.2 cm^−1^ for mono and bi-layer, confirming WS_2_ growth^30^. In the multilayer WS_2_ samples, the E^1^_2g_ and A_1g_ modes were slightly blue-shifted to 352.5 and 421.3 cm^−1^, consistent with values reported in literature^30^. As anticipated, the intensities of the two Raman peaks decreased with the number of layers, and the difference was small while not proportional to the number of layers. The frequency difference between the two modes for multi-layer WS_2_ was smaller than that of mono or bi-layer WS_2_. All of these effects are consistent with previous reports^31^ and support the conclusion that our growth products are WS_2_. Figure 3b presents the PL spectra of mono and bi-layer WS_2_ samples. As observed by other groups, the PL intensity for monolayer samples was much higher than other two samples (bi or multi-layer)^32^. The inset of Fig. 3b shows the PL peaks of a bi-layer sample magnified to be visible, still measurable though appearing as flat when plotted alongside the monolayer sample. It is difficult to observe the PL peak of multi-layer WS_2_ because the band gap changes from direct to indirect when the number of WS_2_ layers changes from mono to multi-layer^33^. The center of the PL peak for mono and bi-layer samples was at 641.4 nm, corresponding to 1.93 eV, similar to values reported in literatures^29^,^34^,^35^. Furthermore, the sharp and intense PL peak indicates the high quality of the WS_2_ monolayer. While the measured FWHM value of the PL peak from exfoliated WS_2_ was 75 meV, the measured FWHM of the PL peak from our CVD-grown WS_2_ was 40 meV, which is comparable to high quality CVD-grown WS_2_ reported in other literature if not better^36^. Figure 3c shows the 2D x-ray diffraction data. The sharp and bright point indicates that the film was well crystallized. Comparing the data to powder diffraction data calculated from a WS_2_ model using Mercury, the q value corresponds to the (004) plane.

**Figure 4:**
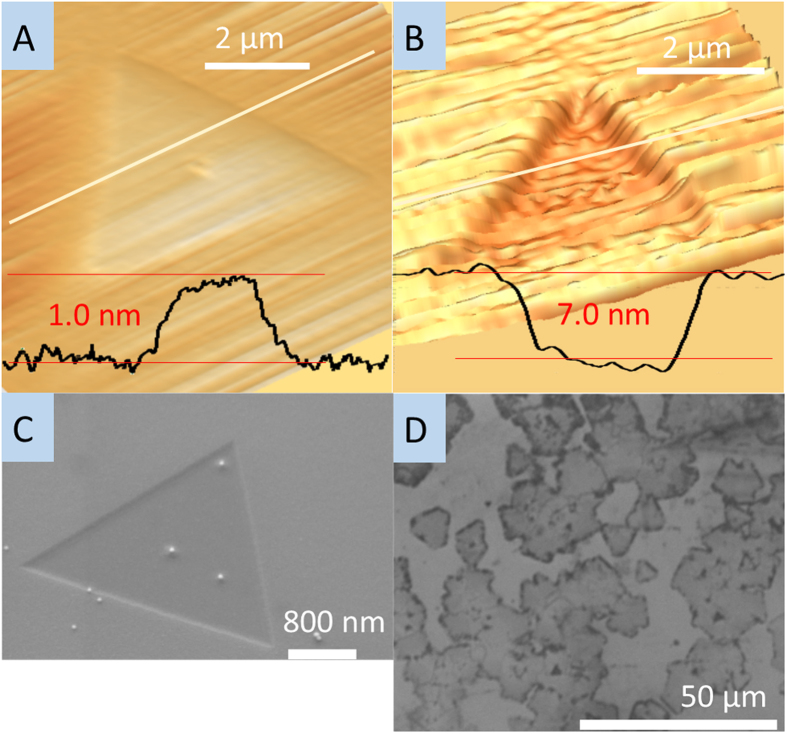
AFM and SEM images of WS_2_ monolayers. (**a**) AFM image of WS_2_ monolayer, (**b**) AFM image of indented WS_2_ monolayer. (**c**) SEM image of indented WS_2_ monolayer. The scale bar is 200 nm. (**d**) SEM image of an etched WS_2_ monolayer by a high concentration of hydrogen during growth. The scale bar is 50 μm.

The AFM and SEM images in Fig. 4 give further information on the effect of H_2_ on crystal growth. There are two etching modes: substrate etching at low H_2_ concentrations and WS_2_ etching at high H_2_ concentrations. In our experiment, we used a WO_3_-deposited SiO_2_/Si substrate as a tungsten source which was placed on top of a clean SiO_2_/Si substrate as a growth substrate. Also considering the presence or lack of Ar, we obtained four different types of WS_2_ deposition samples: either the top (i.e., WO_3_ deposited substrate) or bottom (i.e., oxidized Si substrate), and either with or without Ar flow during growth. Figure 4a depicts an AFM image of a WS_2_ monolayer grown on the bottom substrate with no Ar. The step height measurement of 1 nm gives further evidence of monolayer growth. Figure 4b,c show AFM and SEM images of a WS_2_ monolayer on the bottom substrate grown under a H_2_/Ar mixture. These images show the WS_2_ grown in a 6 nm etched pit, indicating that WS_2_ was deposited only after SiO_2_ substrate etching. We observed the etched SiO_2_ only on the bottom (growth) sample grown in the combination H_2_ and Ar environment. We did not observe SiO_2_ etching in any of the other three sample types, including the top (source) substrate during the same growth as the indented sample. This phenomena can be explained by considering the reduction and sulfurization process of WO_3_. When the H_2_/Ar mixture gas is used for WS_2_ deposition, the concentration of H_2_ gas is not high enough, resulting in the WO_3_ not being fully reduced before sulfur gas is supplied. Then the clean bottom SiO_2_/Si substrate can be etched by a chemical which includes hydrogen, oxygen and sulfur, of which there are a few in the literature known to etch SiO_2_^37^, while the top substrate is protected by the remaining WO_3_. Only when the WO_3_ is completely reduced can WS_2_ growth begin on the top source substrate. Thus, Ar dilutes the hydrogen, in turn increasing available oxygen during the time of sulfur delivery. When only H_2_ gas is used, the high concentration of hydrogen fully reduces the WO_3_ before sulfur is provided. However, as a second effect, when the flow rate of H_2_ was high, above 60 sccm, the monolayer WS_2_ deposited during the growth period was etched to the substrate surface nonuniformly. Figure 4d shows an etched WS_2_ monolayer, located near the edge of the substrate. Comparing images of WS_2_ growth under different hydrogen concentrations supports the explanation that the residual H_2_ causes etching of the already grown WS_2_ crystals after all the sulfur powder has evaporated. This is shown by the WS_2_ being etched more at the edge of the chip, where the H_2_ concentration is the highest due to our sandwich configuration. Also, the boundary of the original single crystal WS_2_ monolayer can be observed due to incomplete etching, showing that the crystal was grown and later etched.

**Figure 5:**
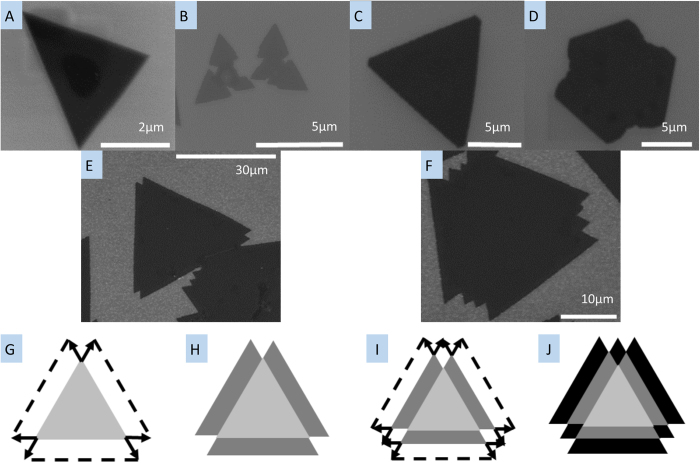
SEM images showing the several growth formations of WS_2_ monolayers. The black triangle shapes are monolayer WS_2_. (**a**) growth formations of WS_2_ monolayers deposited under 30 sccm H_2_ with 100 sccm Ar flow rate, (**b,c**) deposited under 60 sccm H_2_ with 100 sccm Ar flow rate, (**d–f**) deposited under only H_2_ without Ar. (**g–j**) show schematics of the temporal evolution in a sequential growth formation: (**g**) first enlargement step after the termination of the first WS_2_ monolayer growth (**h**) termination of the first enlargement step (**i**) second enlargement step after the termination of the first enlargement step (**j**) termination of the second enlargement step. The scale bar of (**a**) is 2 μm, (**b–d**) are 5 μm, (**e**) is 30 μm and (**f**) is 10 μm.

**Figure 6:**
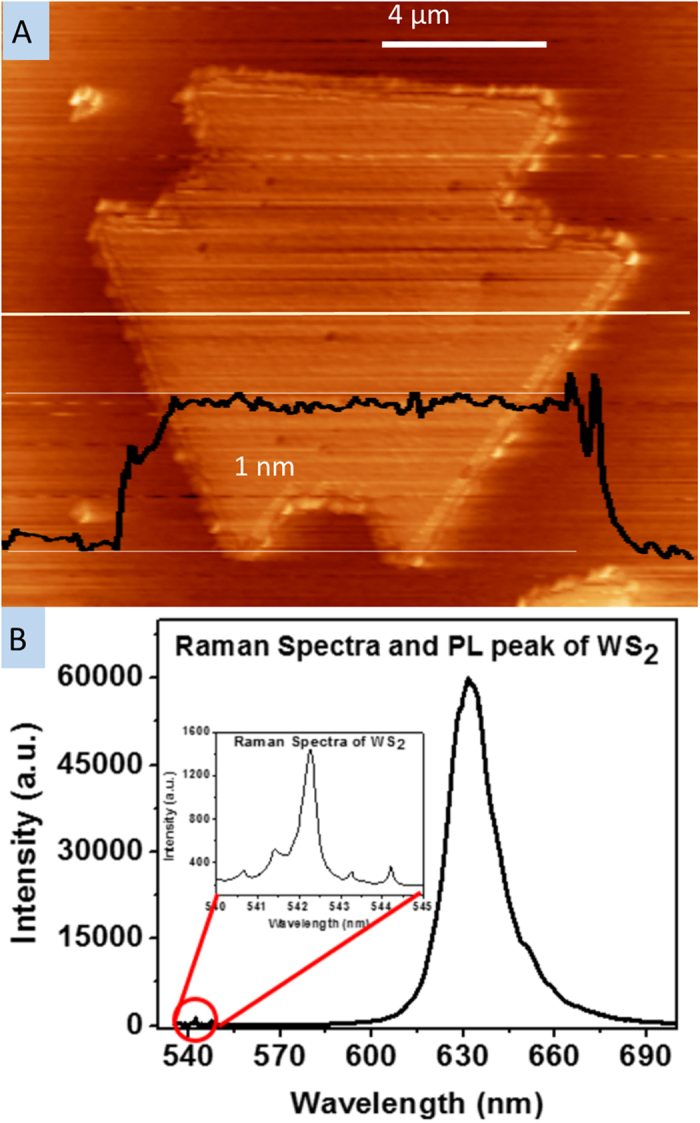
AFM, Raman, and PL of a step-growth sample. The AFM scan (**a**) shows that the sequential growth is in-plane with no break between the crystals. The height of the 630 nm PL peak relative to the Raman signal shows that the crystal is monolayer WS_2_ (**b**).

In our experiment, several growth formations for monolayer samples were observed, and the formations were also found to depend on the concentration of H_2_. Figure 5 shows the different growth processes and the resulting morphology differences. Figure 5a–c show different growth formations grown in an H_2_/Ar mixture environment. Figure 5a shows WS_2_ monolayer growth under low H_2_/Ar flow rate conditions, where all the WS_2_ monolayers are a clear triangle shape and less than 5 μm in side length. Figure 5b shows monolayer growth under high H_2_/Ar flow rate, where we can observe a barb shaped triangle growth process, observed and explained by Cong *et al.*^38^. Figure 5c is a well-known 2D material growth mode, observable when a hexagonal crystal structure has different growth rates on alternate faces^39^. When we supplied only H_2_, we observed two different growth processes, shown in Fig. 5d–f. The growth shown in Fig. 5e,f is a new growth mode not reported in literature to our knowledge. Our interpretation on the temporal evolution of a single crystal of WS_2_ is illustrated in Fig. 5g–j, showing the in-plane sequential growth, creating the multi-apex WS_2_ triangle shape. The growth directions at the corners are shown by the solid arrows, while the dashed lines show the outline of the overall expansion. In this growth, W atoms are abundant on the SiO_2_ surface due to the reduction of WO_3_ by H_2_, as the sample is deposited under a high concentration of H_2_. We attribute this unique formation of the multi-apex WS_2_ triangle shape to the ratio between W and S during the growth; the WS_2_ monolayer becomes a triangle shape when the ratio of W to S is higher than 1:2^40^. In that paper, Wang *et al.* demonstrated that only for reactant delivery in the ratio 1:2 will hexagonal crystals grow, otherwise growth of three faces will be faster than the other three and triangles will form. In the terminated WS_2_ monolayer (Fig. 5a), each side of the terminated triangle of WS_2_ is an active site for in-plane expansion of the monolayer crystal. Figure 5h shows the schematic after the termination of the first expansion, forming an additional apex in each side of the triangle, of which growth state is shown in Fig. 5e. Figure 5i shows a second expansion step and Fig. 5j depicts the terminated second expansion process. Figure 5f is an SEM image after the third expansion process. This process repeats to enlarge the size of WS_2_ monolayers. Figure 6 shows an AFM scan and Raman and PL for a typical step-growth sample. The Raman and PL signatures were taken on the area grown during the first enlargement step after the termination of the first WS_2_ monolayer growth. The AFM scan shows that the growth is in-plane with matched thickness for the subsequent step, and the strong PL signal confirms that it is monolayer growth.

## Conclusion

We have demonstrated large scale WS_2_ monolayer deposition, with up to 433 μm^2^ single crystals and mm^2^ size continuous polycrystalline films. We have furthermore elucidated the effect of the concentration of H_2_ during the reduction and sulfurization process; the relatively simple relationship between increasing domain size with H_2_ flow rate as well as substrate and sample etching and its effect on growth morphology. We have shown that controlling H_2_ concentration is crucial for large area WS_2_ deposition. In the presence of an Ar carrier gas, increasing the local pressure, the crystal size varied relatively little (a few micrometers) between low and high flow rates. In a H_2_ only environment, the flow rate had a dramatic effect on growth. In addition, in the conditions of a high concentration of H_2_ and a low concentration of sulfur gas, the grown WS_2_ was etched. Raman spectroscopy and AFM images confirm monolayer growth in accordance with other groups’ findings. XRD measurement confirms the grown WS_2_ is well crystallized with the (004) plane normal to the substrate. Finally, we have offered an explanation for a new growth mode for WS_2_ single crystal monolayers which works in stages.

## Methods

5 nm thick WO_3_ was evaporated from pellets onto a source substrate which was sandwiched with a clean second substrate for growth, with no space. The sandwiched sample was loaded into the middle of 3′′ quartz tube. For sulfur, we adopted the commonly published method for MoS_2_ and WS_2_ growth of placing solid sulfur powder in the furnace tube upstream of the growth area. The ambient gas was purged out by mechanical pump to the base pressure of 850 mTorr. As the furnace was ramped in temperature at 15 °C/min, the reaction proceeded by reduction of WO_3_ by hydrogen and subsequent sulfurization of the WO_3_. The growth temperature was 900 °C. Ar gas was introduced from 150 °C to reduce moisture and ambient gas and H_2_ gas was supplied from 650 °C (increasing temperature) to 700 °C (decreasing temperature). The deposition pressure depends on the gas type and amount of flow rate. The best result was obtained at 4.5 Torr deposition pressure under 60 sccm H_2_ flow rate. The reduction and sulfurization reactions require a higher temperature than the sulfur evaporation. By placing the sulfur at different places outside of the main heating area of the furnace, it evaporated at different times relative to the substrate temperature. At the optimized location for our furnace setup, the sulfur powder started to evaporate at 830 °C furnace temperature and all sulfur powder was used up after about 30 minutes.

## Additional Information

**How to cite this article**: Kang, K. N. *et al.* The growth scale and kinetics of WS_2_ monolayers under varying H_2_ concentration. *Sci. Rep.* **5**, 13205; doi: 10.1038/srep13205 (2015).
